# Rapid serial blinks: An index of temporally increased cognitive load

**DOI:** 10.1371/journal.pone.0225897

**Published:** 2019-12-02

**Authors:** Ryota Nomura, Shunichi Maruno

**Affiliations:** 1 Faculty of Education and Psychology, Kagoshima Immaculate Heart University, Kagoshima, Japan; 2 Graduate School of Human-Environment Studies, Kyushu University, Fukuoka, Japan; Tongii University, CHINA

## Abstract

In recent years, natural viewing settings with video presentation have been used in neurological and psychological experiments. However, the experienced cognitive load may differ among participants. In this study, we show that rapid serial blinks (RSB) can indicate temporally increased cognitive load with high temporal resolution. We proposed a method to create a personal criterion for respective participants by using empirical blink intervals. When we focused on more than four serial blinks (i.e., three inter-blink intervals), an increased number of RSB detect participants who felt hard to understanding, indicating a poor understanding of the subject matter. By contrast, a constant criterion across participants used in previous study could not detect participant’s understanding. These results suggest that individual differences in cognitive trait of each participant may skew the results of experiments. To avoid biases, we recommend researchers to perform an operational check on individually different temporally increased cognitive loads among experimental groups.

## Introduction

In the field of neurology and psychology, researchers often used controlled experimental settings. Although these researches have provided important findings on neural basis and behavioral tendencies, the stimuli used in those experiments were simple for exploring perception or judgment in more complicated situations where we usually meet in real life. In recent years, natural viewing setting paradigm has been proposed as an alternative method [1–5], providing findings on cognitive activities in more context-dependent environments. When we used videos in experiment, however, it is not obvious to assume that the same video influences on each participant equivalently. Rather, stimulus values may vary participant to participants in natural viewing settings. Hence, we must consider the differences of participants’ experience during watching the stimulus video. Especially, in the cases the experimental procedures include cognitive decisions, it is necessary for researchers to adequately control each participant’ cognitive load in the subsequent analysis.

Cognitive load has been often measured using pupil size because pupil dilation relates to cognitive controls [6] and occupation of working memory [7]. However, the pupil indices are too slow to capture the ongoing cognitive functions because its response peak comes 1 s after the target onset time. Thus, pupil dilation can be used for cognitive tasks that demand a shift slower than 1 Hz. Although dilation deconvolution of pupil size has been proposed as a method for highly temporally-resolved estimations [8], the calculation cost is still large.

Here, we show that rapid serial blinks (RSB) in natural viewing settings can indicate temporally increased cognitive load with high temporal resolution and low calculation costs. In recent years, some researchers have proposed that characteristics of spontaneous blinks relates to individual differences in cognitive performances. In a resting situation, spontaneous blink rates variability predicts the performance on the subsequent IQ test [9]. In experiments, blink rates immediately increase after finishing cognitive tasks while blinks are suppressed during cognitive events. In the experiments, the number of sequential blinks, referred as “bursts” or “flurries”, correlates with the degree of cognitive load [10]. Most recently, a study revealed that human can learn to use strategic blinking to maximize obtained visual information under the constraint to keep humidity of eye surfaces [11]. Therefore, blink rates are sensitive to cognitive demands rather than they vary in arbitrary way [12]. In natural viewing settings as well, temporal increases of blink rates would be an index to detect occurrences of increased cognitive load. If we can adopt Nomura et al.’s computational approach [13], which can reproduce four major distributions of inter-blink intervals, assuming cognitive loads accumulate in each cognitive event. When the integrated cognitive loads reach a threshold, a blink generator in the nerve system immediately elicits a blink. Hence, we assume that RSB occur due to increased cognitive load at a rapid pace, leading the integrated cognitive loads repeatedly reaching the threshold within a few seconds.

In the current study, we rigorously controlled the probability of cognitive load by providing two video-recorded lectures on mathematics: A lecture on multiplication of a two-digit and three-digit number—hence called the multiplication video—and a lecture on differentiating log(x)—hence called the differentiation video. In the multiplication video, we can assume that temporally increased cognitive loads would occur less often among all participant groups. In the differentiation video of log(x), on the other hand, temporally increased cognitive loads would occur more frequently but depending on the participants’ levels of understanding because participants’ experienced difficulty vary depending on the each participant’s ability and knowledge of mathematics. We therefore observed the participants’ blinking behaviors and examined whether we could predict the numbers of RSB by the participant’s understanding level.

However, substantial differences would exist among people’s physical blinking characteristics, such as those of the nerve systems and orbicularis oculi muscles, which yield various blink parameters such as eyelid closing speeds and blink depths. Thus, blink rates vary in accordance with personal factors such as age, smoking, and dry eyes [14]. In the present study, we adopt individual criteria and propose a statistical model considering differences in total number of blinks.

First, we applied personally determined criteria of statistically sufficient short-term serial blinks to an individual’s blinking time series. The personal threshold of serial blinks is defined as the time duration corresponding to the lower 2.5% of the distribution of shortest intervals, which was estimated using randomly shuffled surrogate data of unitary or sequential inter-blink intervals (IBI^(*m*)^, *m* = 1, 2,…, 10, for detail, see Method).

Second, we consider the total number of blinks as a control variable. If the total duration of serial blinks in actual data is less than the personal threshold, such a set of blinks were regarded as RSB. Thus, the number of RSB may depend on the participants’ increasing tendency of blinks. In other words, RSB are more likely to occur if the individual shows a higher total number of blinks. Hence, we use the total number of blinks as an offset term in the linear model to predict the number of RSB. In addition to this, we consider the durations of videos as the second offset term. The predictive equation is as follows:

$$\log\frac{\#RSB}{\#B_{k\;total}\times t_{video}}=\beta_{0}+\beta_{1}\;u,$$ (1)

where #*RSB* is the mean number of RSB and #*B*_k *total*_ is the total number of blinks for participant *k* (*k* = 1, 2, …, *n*) and variable *u* (*u* = 0, 1, 2) represent the participant groups determined by the understanding level. Considering the exponential function, Eq (1) can be mathematically transformed into

$$\#\mathrm{RSB}={exp(\beta }_{0}+\beta_{1}\;u)\times \#B_{k\;total}\times t_{video}$$ (2)

where the first offset term is the number of blinks #**B**_**k total**_, which adjusts the differences in the total number of blinks among participants [15]. Then, the second offset term **t**_**video**_ is the length of videos for each condition. Thus, Eq (2) means that the number of RSB is an exponential function of participant groups considering an individual’s total number of blinks and durations of videos. Because the number of RSB is discrete, a generalized linear model (GLM) for count data was applied. To estimate the slope and intercept, we used negative binomial distributions as the error structure of all RSB because the variances were larger than the means, which is called as overdispersion. In such situations, a Poisson distribution has limited ability to yield accurate estimations because in a Poisson distribution the mean is equal to the variance. For data with overdispersion, a negative binominal distribution is adequate because the mean may be unequal to the variance in this distribution.

## Material and methods

### Participants

Complete data were obtained from 20 participants (11 male and 9 female). All of the participants had previously taken basic mathematical classes (Courses I and Course II), which included logarithmic calculations, and a portion of the participants had also taken an advanced mathematical class (Course III), which included calculus, in high school. These participants were categorized as having low (six participants), middle (seven participants), and high (seven participants) understanding levels based on the results of a post-video test regarding the differentiation of log(x): The participants with incorrect answers belonged to the low group. Correctly-answered participants were assigned to the high group if they believed the provability of correct answer as 100% in their written response. The remaining participants were categorized as the middle group. Apart from these data, the data obtained from one participant was excluded from the analysis because there were several instances in which this participant’s eyelids closed for more than 15 s, suggesting that the participant shut their eyes intentionally.

### Videos

The lectures were performed by one male cram school teacher (24 years old, 5 years’ experience). Before the video recording, the experimenter and the teacher had a meeting to confirm the logical consistency of the lectures. To select the content of the lecture video for the main experiment, a pilot test was conducted. In this pilot test, sixteen undergraduate and graduate students, who were a completely different group from the participants in the following main experiment, participated in a written test comprising 10 questions about the differentiation of functions. All participants had learned basic classes of mathematics (Course I and II) and eight of the participants had taken advanced mathematics classes (Course III) in high school. The mean scores were 8.96 and 1.89 for experienced and non-experienced students, respectively. In the list of mathematical questions, the differentiation of log(x) showed the highest discriminative power; eight (100%) experienced (Course I, II, and III) and two (25%) non-experienced (only Course I and II) students reached the correct answer. In the main experiment, multiplication video is used to measure base-line blink rates for each participant.

### Procedure

All participants were separately invited to enter a laboratory room. Each participant sat on a chair approximately 60 cm away from the monitor (1920 × 1080, BenQ, GW2470H). Toward participants, the experimenter briefly explained the aim and the procedure of this experiment. In this phase, however, to avoid the participants’ intentional control of blinking, the experimenter told the participants that “this experiment aims to measure where you look on the monitor while you are viewing math lecture videos.” All participants agreed with participating this experiment. Then, the participant took a pre-video test including four questions about basic differentiation.

After calibrating an eye-tracking device, the participant was instructed “to watch videos to understand the content, which was followed by a post-video test.” To measure the baseline for RSB, all participants watched the multiplication video first. While the participants were watching each video, the experimenter left the laboratory room. After finishing the video presentations, the experimenter re-entered the room. All participants attended a pen-and-pencil post-video test. At the same time, the participants were asked to rate the subjective difficulty to understand the both videos, using a six-point Likert scale. Then, the experimenter explained that the actual purpose of the experiment was to measure the timings and frequency of eyeblinks while watching the lecture videos. No participants withdrew their agreement with participating this experiment. The participants also gave permissions for their eye information to be used in the study.

This study was carried out in accordance with the recommendations of Ethical Guidelines for Medical and Health Research Involving Human Subjects, Ministry of Health, Labor and Welfare, Japan. The protocol was approved by the Ethical Committee of The University of Tokyo. All subjects gave written informed consent in accordance with the Declaration of Helsinki.

### Data collection and definition of RSB

Blink timings were detected using an eye-tracking device, The Eye Tribe (The Eye Tribe, Copenhagen, 30 Hz). Previous research has utilized optical measurements of blinks [9]. In this study, blink timing was operationally defined as the disappearance of pupils for serial three frames due to the eyelids, while the face of the participant was detected (for detail see, Ref. [16]). The complete data is shown in the Supporting Information (S1 Table).

In previous study [17], “bursts” of blinks were judged using a constant value: More than two blinks, of which blink intervals were less than 0.5 s. For comparison, we calculated blink bursts (BB) using this criterion as well. If the individually different criteria can predict the understanding groups than the constant criterion, our proposed method would be more validate.

**Fig 1:**
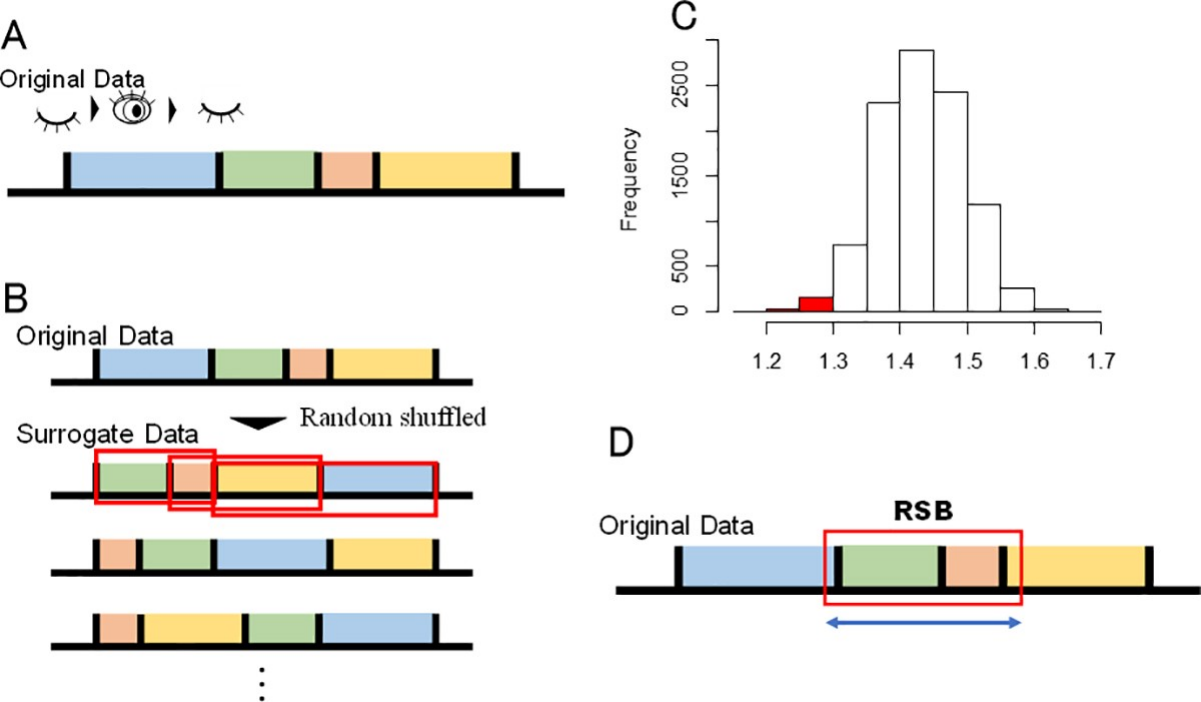
Schematic illustrations for the calculation of personal criteria. Each *m* (*m* = 1, 2, 3, 4) in IBI^(*m*)^ shows the number of IBI used for creating personal criteria. This figure shows the case of IBI^(2)^, thus the two serial intervals are used for calculation. (A) Measuring IBIs, (B) making surrogate data (10,000 cases) by randomly shuffling the experimental data and calculating durations of serial intervals, (C) calculating the lower 2.5% of a distribution of unitary or sequential IBIs obtained by the procedure (B), and (D) applying the calculated criterion and detecting RSB.

The RSB for respective participants were defined as the serial blinks of which total intervals were shorter than statistically sufficient short-term personal criteria. Fig 1 shows the procedure to determine the criteria. The personal criteria for IBI^(*m*)^, where *m* is the number of used interval(s), were calculated as follows:

First, to cancel temporal correlations, we randomly shuffled participant’s all IBI obtained during the experiment within person. Then, we obtained a distribution of *m*-length serial IBI which is calculated as sequentially summations of serial IBI for 1, 2, …, *m*, in each randomly shuffled IBI. Next, we recorded the lower 5% point of this distribution. By repeat this procedure for 10,000 times, we then obtain a normal distribution of the lower 5% points of *m*-length serial IBI, owing to central limit theorem. Finally, we used 95% confidence intervals of this normal distribution as the criteria. In other words, we adopt lower 2.5% point as the criterion of statistically short enough interval for this *m*-length serial IBI. We call this personal criterion as IBI^(*m*)^. If an observed total interval of *m*-length serial blinks is shorter than IBI^(*m*)^, we regarded the blinks as RSB. In this study, we calculated unitary (*m* = 1, i.e., simple random shuffle) or sequential IBIs (*m* = 2–10, i.e., random shuffle and summation of *m*-length serial blinks). For all analyses in this study, we used R 3.3.2 with MASS package.

## Results

### Operational check

First, to verify the validity of the experimental manipulation, we present the summary of the participants’ responses to the two lecture videos. The range of the total number of blinks was 13 to 166 (SD = 41.69, Mean = 68.95, Median = 68) for the multiplication videos and that was 31 to 270 (SD = 60.01, Mean = 122.3, Median = 127.5) for the differentiation video. Likewise the previous studies [13–14], the total number of blinks in this paper demonstrated substantial individual differences. Moreover, simple counts of blinking could not detect the understanding level (*z* = −1.73, *p* = 0.08 for the multiplication videos; *z* = −1.31, *p* = 0.19 the differentiation videos).

The subjective difficulties evaluated by the high, middle, and low participant group were 1–2, 3–4, and 5–6, respectively. This means that the participant groups determined by post-video test of log(x) were corresponded to the ratings of participant’s subjective difficulty of the differentiation video. In regard to the multiplication video, on the other hand, the group-wise participant’s subjective difficulty scores were 1.0 to 1.5, indicating that there was no difference among participant groups.

**Fig 2:**
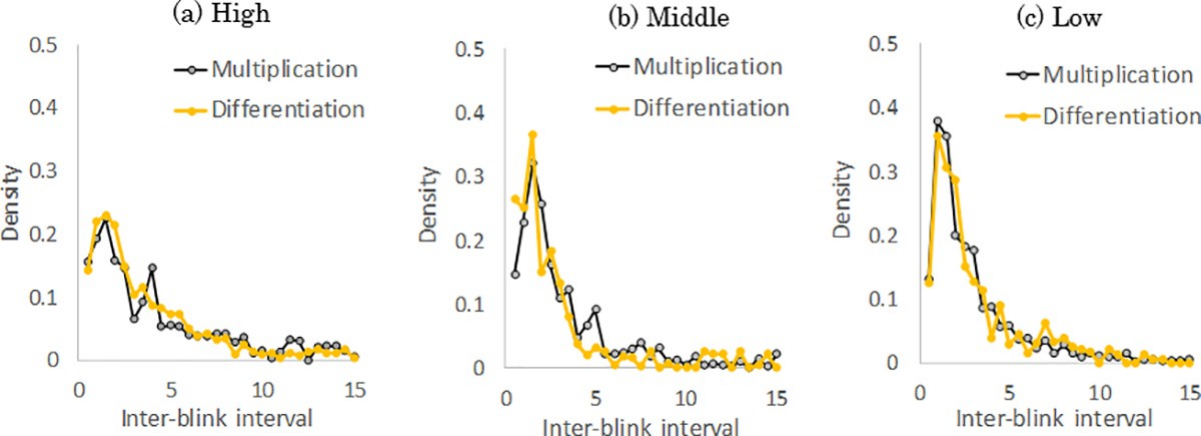
The distributions of IBIs for each group while high, middle, and low understanding participant groups watched the multiplication and differentiation videos.

Then, to compare the features of IBIs among groups, we calculated distributions of IBIs obtained while viewing the two videos, respectively. Fig 2 shows the distributions of IBIs for high, middle, and low understanding participant groups who watched the multiplication and differentiation videos. As shown in Fig 2, high understanding participants group showed a lower peak at 1–2 s compared to other two groups. However, in all groups, the distributions of IBIs demonstrated a nearly similar shape for each video. This indicates that the distributions could not explain the differences in blinking patterns corresponding to the videos when we do not consider temporal information.

### IBIs that detect the understanding level of groups

**Fig 3:**
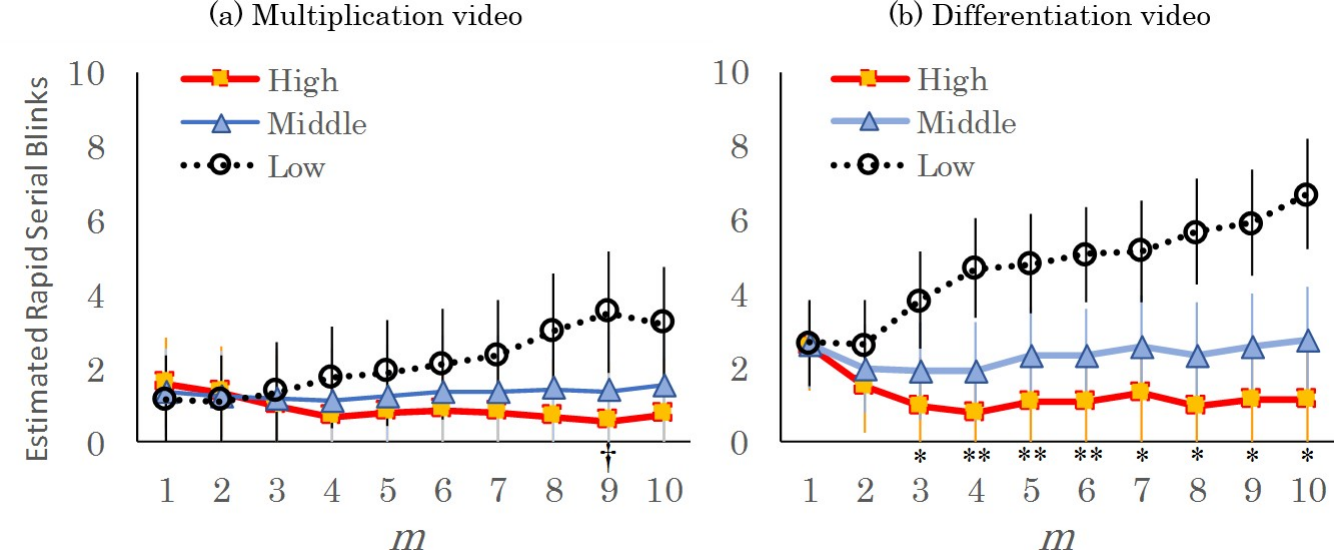
The estimated numbers of RSB for IBI^(m)^ vs. understanding level (High, Middle, and Low). IBI^(*m*)^ means that *m*-length serial blinks were used for calculating the criterion. A generalized linear model (GLM) for count data was applied. To estimate the slope and intercept, we used negative binomial distribution as the error structure. The points show the mean values calculated using the means of total blinks (differentiation 122.3 times; multiplication 68.95 times) and video duration (differentiation 397 s; multiplication 209 s). The error bars show the exponential standard errors of the estimated RSB. †*p* < .10, *p < 0.5, and **p < .01.

RSB were detected by using the personal criteria (see, Method). Fig 3 shows the estimated counts of RSB for each understanding participant group using the offset terms, mean numbers of blinks and video durations. For instance, regarding IBI^(4)^ in Fig 3(A), total number of RSB for high understanding group was estimated as #RSB = exp(−9.2462 + −0.8973 * 2) × 122.3 × 397 = 0.78. Likewise, IBI^(4)^ for middle understanding group was estimated as exp(−9.2462 + −0.8973 * 1) × 122.3 × 397 = 1.91 and IBI^(4)^ for low understanding group was estimated as exp(−9.2462 + −0.8973 * 0) × 122.3 × 397 = 4.68. As shown in Fig 3(B), we can find differences in the number of RSB for the low understanding group only for the differentiation video, in the case we use a criterion of more than three IBIs. Addition to this, the predictive power was most large when we use IBI^(4)^ (*z* = −3.09, *p* = 0.002). In regard to the multiplication video (Fig 3(A)), by contrast, no significant differences in RSB were found among the groups in all IBI^(*m*)^, under the conditions of *m* = 1–10.

To test the validity of the proposed criteria for RSB, we compare these results to BB based on the across-participants criteria proposed in a prior study [17]. When we adopt a constant criterion among all participants, consistent results relative to the understanding level were not demonstrated: No significant differences were evident in the number of BB: *p* = .338 and *p* = .701 for the multiplication and differentiation videos, respectively.

### Temporal locations of RSB

**Fig 4:**
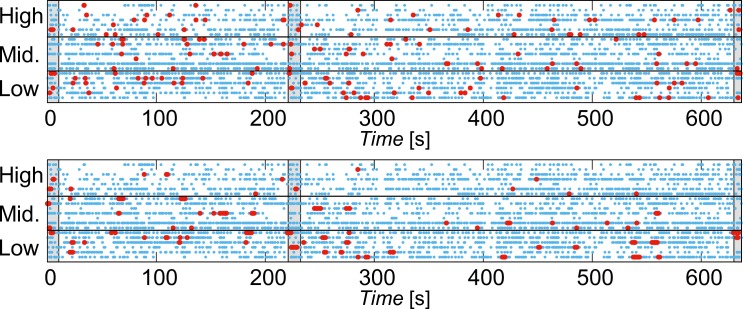
**Raster plots of blink occurrence and RSB for (a) IBI**^**(1)**^ **(top panel) and (b) IBI**^**(4)**^ **(bottom panel).** Light blue points show the temporal locations of blinks and red points show the detected RSB. The gray rectangles in each panel indicate durations in which participants viewed the black screen between the video presentations. The multiplication video was presented from 10.5 s to 220 s and the differentiation video was presented from 232.5 s to 630 s.

With using RSB, we estimated the temporal locations where high cognitive load was elicited. Fig 4 is a raster plot that show the temporal locations of blinks (light blue points) and RSB (red points). The top lane of each panels show the blinking patterns of audiences who are high understanding group.

**Fig 5:**
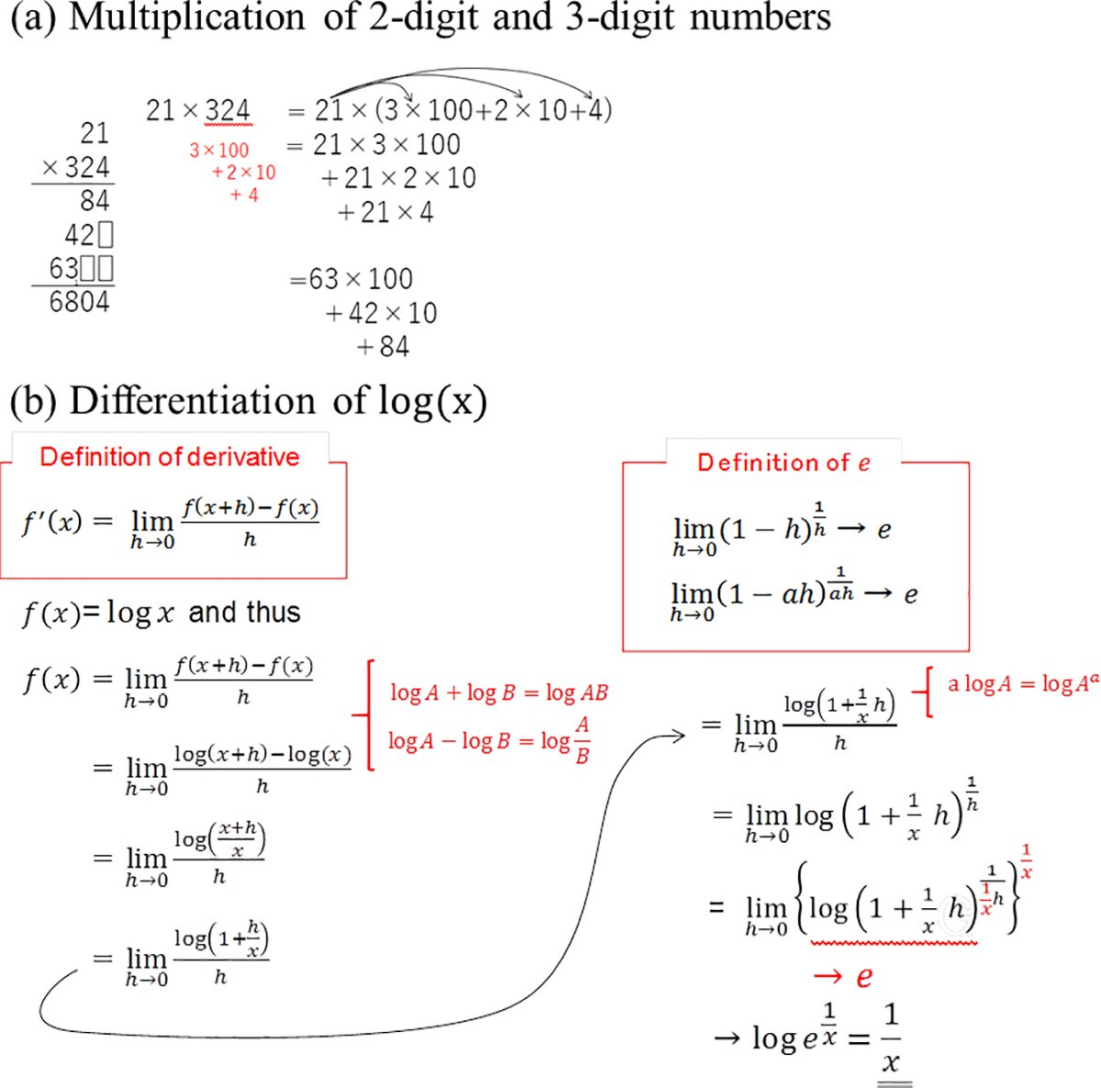
The contents written on the blackboard at the end of each lecture. (a) The top panel shows the contents of the multiplication lecture and (b) the bottom panel shows the contents of the differentiation lecture.

The center and bottom lanes demonstrate that of middle and low understanding participant groups, respectively. As shown in the bottom panel, when we used IBI^(1)^, i.e., simple random shuffle IBIs, RSB appeared across high, middle, and low understanding participant groups throughout the experiments. There were no difference in the density of RSB among groups. However, when we used IBI^(4)^, the density of RSB among the low understanding participant group was higher than that among the high understanding participant group during the differentiation video after 232.5 s (Fig 4(B)). Moreover, the occurrence timing of RSB overlapped among participants who categorized into middle and low understanding participant group at e.g., 540 s and 550 s. These time locations corresponded to the transformation of fraction into exponent presentation of 1/h (Fig 5(B), just below the “Definition of e”).

## Discussion

When we focused on more than four blinks (i.e., three IBIs), the number of RSB increased for lesser understanding participants watching the differentiation video, while no group difference was found when watching the multiplication video. This suggests that we can identify the probability of temporally increased cognitive load in natural settings by measuring RSB despite large individual differences in blink parameters. On the other hand, the distributions of IBIs did not change for each video (Fig 2(A)–2(C)). Therefore, temporal information of blinking patterns was crucial for detecting these differences in the probability of temporally increased cognitive load depending on participants’ understanding levels.

Another possible interpretation about the differentiation video setting is that the participants with poor understanding could not comprehend the contents of the lecture at all, and therefore stopped thinking about it. However, this possibility was unlikely because all of the participants had previously attended basic mathematical classes (Course I and Course II) in high school. They remembered basic logarithmic formulae, such as log *A* + log *B* = log *AB*, $\log\;A-\log\;B=\log\;\frac{A}{B}$, and *a*log *A* = log *A*^*a*^ (red-coloured formulae in Fig 5(B)). Besides, these participants’ rating scores of difficulty in understanding the differentiation video were very high, i.e., 5–6 on a scale of 1–6, 6 being felt the most difficult. Hence, it is less plausible that the participants with poor understanding experienced the attention drift away from the task that lead to ‘zero’ cognitive load through inability to understand the contents of the differentiation video.

In previous study, researchers often used blink provability [9] or proportions of blinks [10] to capture the variation of blink patterns during cognitive task. The RSB would be another index of cognitive load during tasks. In the present study, we hypothesized that temporally increased cognitive loads would occur less often among all participant groups for multiplication video. This would be plausible because the subjective rating scores of the difficulty were very low, i.e., 1.0–1.5, for the multiplication video. Nonetheless, not a few RSB of participants were detected in each group during watching the multiplication video, even when we used sensitive index IBI^(4)^. These false positive responses would be elicited because cognitive load is one of several factors that cause increasement of human blinking [10, 14, 18]. However, we would focus on the results that the RSB often occurred for the lower understanding participant group when participants experienced heavy cognitive lord during watching the differentiation video, where participants’ experienced difficulty would be certainly different.

In neurological and psychological experiments that involve a participant’s higher order cognitive functions, the same stimuli may impose various levels of cognitive loads on participants. To avoid unexpected biases, we recommend researchers to perform an operational check on individually different cognitive loads among experimental groups. If the number of RSB were different among participants, researchers can use them as covariance for ANCOVA. In recent years, advances in technology aid all researchers to automatically detect spontaneous blinks [19].

Human blinking characteristics have large interindividual variability [14, 17]. This has been a problematic fact for many researchers from early empirical studies [20] to current days [11,13]. The individual-based criteria proposed in this paper would be one of the resolutions for psychological phenomenon being problematic because of its large interindividual variability. To create an individual-based index would be a standard tool for next generations.

## Appendix A

### Transformation of predictive linear model

The Eq (1) in the main text can be transform to Eq (2) in the main text as follows:

$$\log\frac{\#RSB}{\#B_{k\;total}\times t_{video}}=\beta_{0}+\beta_{1}\;u$$

$$\log\#\mathrm{RSB}-\log\#B_{k\;\mathrm{total}}-\log t_{\mathrm{video}}=\beta_{0}+\beta_{1}\;u$$

$$\log\#\mathrm{RSB}=\beta_{0}+\beta_{1}\;u+\log\#B_{k\;total}+\log t_{video}$$

With taking the exponential function for both sides of the equation,

$$\#\mathrm{RSB}={exp(\beta }_{0}+\beta_{1}\;u+\log\#B_{k\;total}+\log t_{video})$$

$$\#\mathrm{RSB}={exp(\beta }_{0}+\beta_{1}\;u)\times exp(\log\#B_{k\;total})+exp(\log t_{video})$$

$$\#\mathrm{RSB}={exp(\beta }_{0}+\beta_{1}\;u)\times \#B_{k\;total}\times t_{video}$$

## Supporting information

S1 Table Dataset of blink timing. The first column indicates the ID of participants and the second column shows the initiation time of each blinking.
(TXT)
